# An R-based integrated method for producing river bathymetry and cross-sections from recreational-grade sonar sensor data

**DOI:** 10.1016/j.mex.2024.102852

**Published:** 2024-07-06

**Authors:** M. Redana, E. Carrero-Carralero

**Affiliations:** a Department of Zoology, University of Cambridge, Cambridge, UK; b Fluvial Dynamics Research Group (RIUS), Universitat de Lleida (UdL), Catalonia, Lleida 25198, Spain

**Keywords:** Sonar, Water depth, Bathymetry, Cross-section, Interpolation, GAMs, Open source, Sonar data to bathymetry

## Abstract

Water bodies’ bathymetry is a crucial information for understanding and sustainably managing water resources. Bathymetric surveys can be expensive due to sonar equipment cost, but low-cost alternatives options exist. We present a methodology that standardize the bathymetric data collection and processing of recreational-grade sonar data. The sonar data postprocessing if fully implemented in R, with ready to use functions able to produce bathymetric maps or extract river cross sections’ metrics with minimal computing efforts. The method robustly produces a variety of outputs; the performance of the equipment adopted and of the interpolation technique allow for high accuracy and low-cost bathymetric reconstruction.

• The method implemented allow for a robust and consistent processing of recreational-grade sonar water depth measures.
• Through R-based functions the data are postprocessed to obtain bathymetry maps also for complex shape waterbodies.
• Further metrics of rivers/channel cross sections can be extracted.

## Graphical abstract

**Figure:**
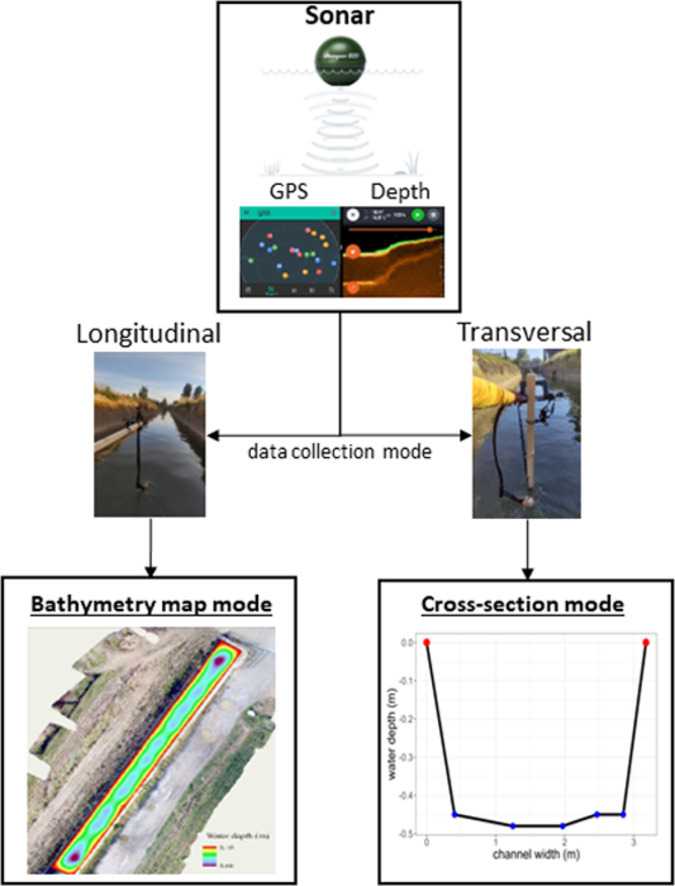

Specifications table

**Table:** 

Subject area:	Environmental Science
More specific subject area:	*Hydrology*
Name of your method:	*Sonar data to bathymetry*
Name and reference of original method:	*NA*
Resource availability:	*Deeper sonar Chirp+2, Android smartphone, R*

## Background

Water bodies’ bathymetry is a crucial information for understanding and sustainably managing water resources. Bathymetry can be reconstructed with *in-situ* or remote sensed measures of water depth. The latter includes the use of LIDAR data or multispectral images (e.g., [1,2]) with the potential to have large spatial coverage; nonetheless, this technique is strongly limited by water depth and turbidity. *In-situ* bathymetric data collection is usually based on bathymetric sonar mounted on vessels; this well-established method provides a high accuracy in the water depth measurement, but it is limited by the cost of the equipment. For relatively shallow water areas (e.g., rivers, ponds and coastal areas), some authors have successfully tested the capability of low-cost and recreational-grade sonars (e.g., fish finder sonar) to collect water depth measures and reconstruct a full bathymetry of the area (e.g., [3]). This approach allows a cost-effective bathymetry survey, especially for low-budget research and monitoring projects. Nonetheless, the low-cost equipment-based methods are still relatively poorly explored; thus, there is a lack in the standardization of bathymetric data acquisition, elaboration and map production from recreational-grade sonar. We developed a methodology that allow the reconstruction of bathymetric maps or river cross-sections, based on a recreational-grade sonar water depth data, specifically the Deeper Chirp+2 (https://v1.deepersonar.com/uk/en_gb). The method here described cover all the phases of the workflow of the bathymetric map production, from data collection to data preparation, interpolation and final validation. Specifically, the data preparation, interpolation and validation are implemented in three R-based functions. The first function (named “deeper_filler”) deal with GNSS position synchronization with water depth data; the second function (named “bathymetry_map”) imports the output of the first function and produce an interpolation using a soap film smoother spline approach through Generalized additive models – GAM [4], that has been proved to be effective for complex morphology/boundary water bodies; the third function (named “cross_section”) can be activated in the case the sonar data has been collected to obtain river cross-sections rather than 2D bathymetric maps. This function allows to interpolate point of river section water depth and estimate the wet perimeter and area. The implementation in R of these three steps will improve the standardization of data elaboration procedure, allowing the use of advanced interpolation techniques and only requiring minimal computing skills.

We summarize here all the methodology, form data collection, to map production and customization, along with the accuracy test results on the sonar and interpolation water depth estimation.

## Method details

### Sonar component

Active Sound Navigation and Ranging (SONAR) are capable of estimating object distance (e.g. riverbed, seafloor, etc.) by emitting sound pulses into the water and computing their returning time to the detector. On the market are now available a number of recreational-grade sonar, mainly developed for fishing purposes. In this method we tested the sonar Deeper Chirp+2 (Fish Deeper) that is compact and portable sonars with an accessible cost (∼300£ at the time of writing). This device is capable, through Wi-Fi connection with any smartphone, of real-time data display (including water depth, stratification, vegetation presence estimation, water temperature) and data storage, exportable by the user in .csv format.

**Fig. 1:**
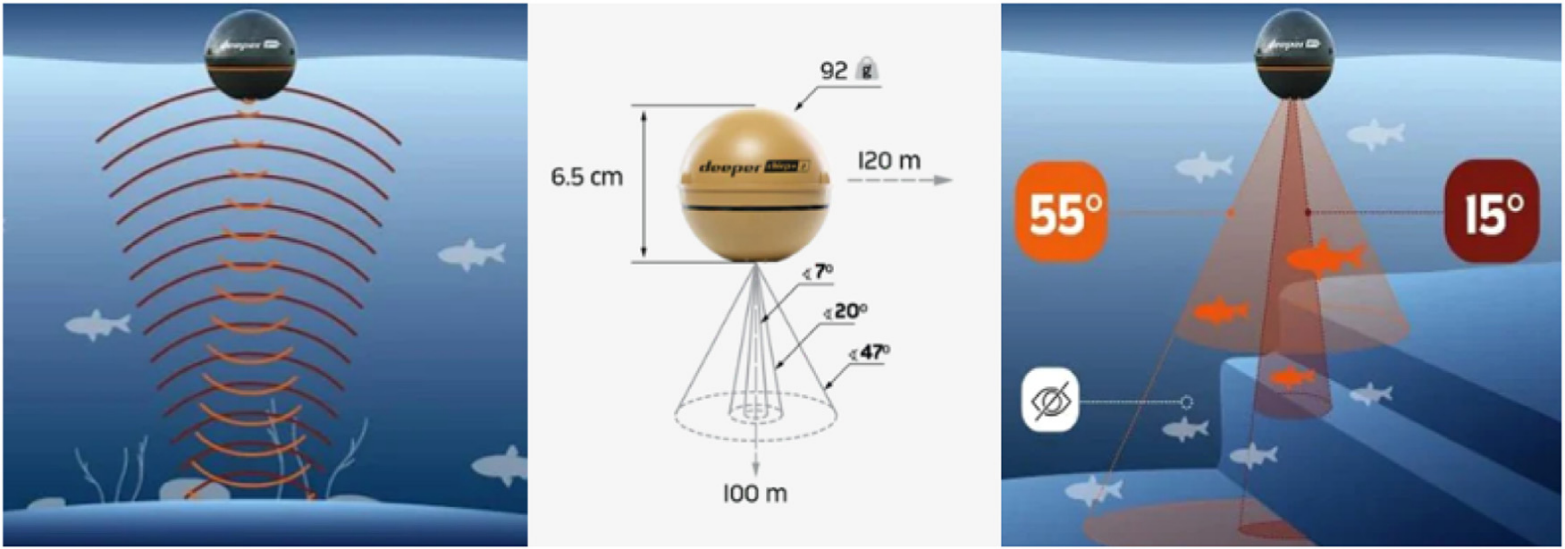
Deeper sonar Chirp+2 technical specifications (https://deepersonar.com/en-all/products/deeper-smart-sonar-Chirp-2).

The Deeper Chirp+2 sonar emits 15 pulses per second (i.e., 15 water depth measures each second), offering three beam angles: Narrow 675 kHz (7°), Medium 240 kHz (20°), and Wide 100 kHz (47°). The cone diameter (CD) size of the sound pulse on the target (Fig. 1) depends on the beam angle chosen by the user and from the water depth (range 0.15–100 m). It can be calculated as:

(1) $$CD=\;2*Water\;Depth*\tan\left(\frac{BeamAngle}{2}\right)$$

**Fig. 2:**
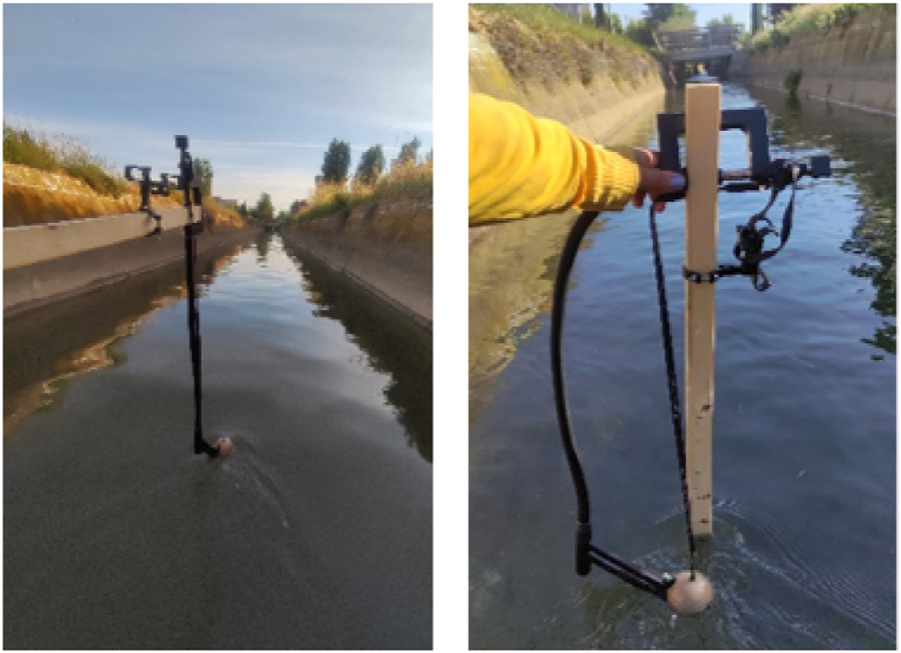
Sonar measurement with flexible arm adapted with different measurement setting.

The Deeper Chirp+2 is equipped with an integrated temperature sensor (−20 °C to 40 °C) and a GNSS module (compatible with GPS, GLONASS, Galileo, and QZSS), ORG1510-MK05, characterized by an update rate of 10 Hz and nominal accuracy <1.6 m in optimal conditions (Origin GPS, https://origingps.com/). The sonar can be coupled with a flexible arm (Fig. 2) that optimize its positioning in the water in different conditions.

Must be noted that an accurate estimation of water depth measure positioning (i.e., latitude and longitude) is essential to produce reliable bathymetric maps. Thus, in case of low or poor quality satellite coverage or where higher accuracy is needed, the sonar built-in GNSS module may be not sufficient to reach the desired level of accuracy. Thus, the sonar can be integrated with independent GNSS data. Specifically, our method allows for the integration of external GNSS rover measurement, as explained in section 2.

### Data collection protocol

The sonar data collection process only requires the Deeper Chirp+2 and a smartphone with the dedicated app installed (Fisher Deep, https://play.google.com/store/apps/details?id=eu.deeper.fishdeeper&hl=en&gl=US). Through the app is possible to establish and control the Wi-Fi connection between sonar and phone (80 m max range, extensible to 150 m), customize the sonar acquisition, including beam angle (see above) and set the acquisition mode. If the user will use the built in sonar, the “Bait Boat” mode or the “Bank” mode must be activated; both modes allow to use the Chirp+2 GNSS module to acquire positioning during the survey.

The data collection workflow initial step is to place the sonar in the water to initialize the Wi-Fi and connect the phone (at this stage can be useful setting the phone into airplane mode to minimize possibility of Wi-Fi connection jumping if other known Wi-Fi network are available in the area). Once the connection is established the sonar will start recording when the minimum water depth is reached (15 cm) and the data displayed on the App. Must be underlined that if the Chirp+2 built-in GNSS is used to determine sonar measure position, the user must ensure a stable GNSS coverage. This involve stabilising the GNSS connection maintaining submerged the sonar for ∼1-minute and cheeking the green GPS signal before starting the actual scanning. Simultaneously the data (water depth, GNSS position, water temperature, timestamp) are automatically stored in a .csv file. Our method offers two possible data elaboration approaches (see section 3):

(I). bathymetry map mode (i.e., full, raster format, bathymetric map)
(II). cross-section mode (i.e., 2D representation of the desired cross-sections and relative area, possibly for flow estimation application).

**Fig. 3:**
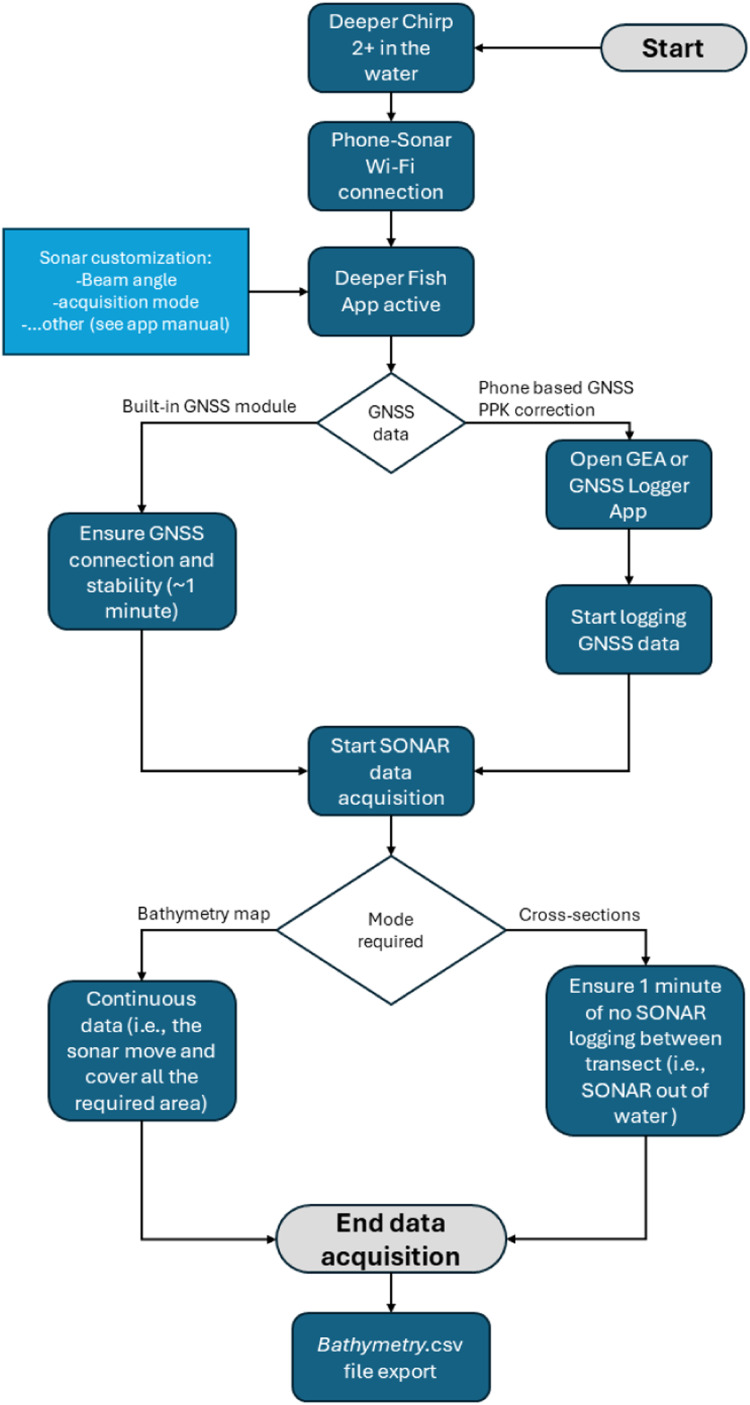
Schematic representation of the protocol for sonar and GNSS data acquisition.

Thus, the user data acquisition path must be organized based on the desired output (see section 3). In the case of the bathymetry map mode the motion of the sonar can be continuous in the water and potentially homogeneously cover all the required area. In the case of the cross-section mode the only required data is a transect of the required (river) section; if there are multiple sections the user must ensure a minimum one-minute gap between each transect where the sonar is outside the water (this is to ensure the algorithm in 3.2, correctly disentangle the subsequent transects). Further, still in case of multiple transects, if the GNSS data are being collected with the Chirp+2 built-in module, the user must ensure that the GNSS connection is again stable after every 1-minute gap between transects. In the cross-section mode the same transect can be repeated several time (no interruption required) to improve accuracy (see section 3.1) and the distance between river bank and first/last sonar measure (minimum water depth of 15 cm for sonar readings) must be noted. Given the specifications of the built in ORG1510-MK05 GNSS receiver (see section 1), if it is required a higher accuracy in the sonar point positioning estimation, is it possible to couple the sonar with other GNSS receiver and antenna, potentially able to acquire raw GNSS data, obtaining RINEX files. These can be further corrected with post-processing kinematic (PPK), potentially reaching centimetres-level accuracy if a nearby base station is available. It is not the focus of this work to list methods and equipment for PPK; nonetheless, it's worth to be underlined that recently several android devices (i.e., smartphones) are capable of raw GNSS data release, turning them in low-cost GNSS receiver devices [5]. The acquisition of raw GNSS data is feasible through already existing app such as Open GEA [6] or GNSS logger. In our methodology both sonar data geolocation paths (i.e., with the Deeper Chirp+2 GNSS module or with external rover) are possible. In Fig. 3 is schematized the data acquisition workflow.

**Fig. 4:**
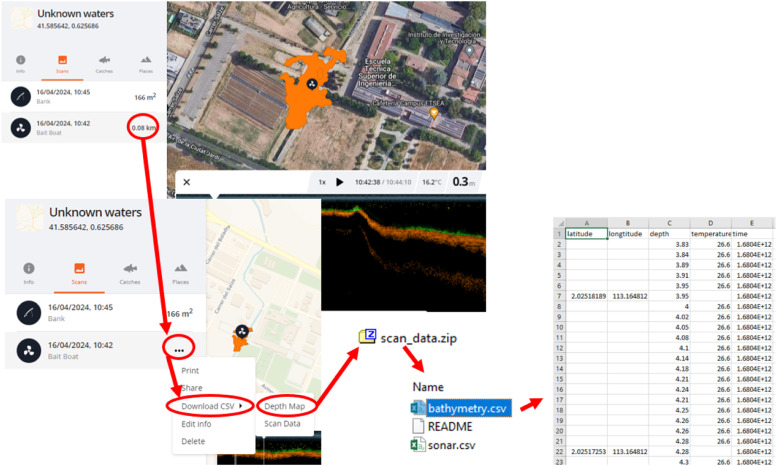
Steps to follow to recover measured sonar data and produce the “bathymetry.csv” file.

During the acquisition, the sonar data are stored locally in the phone and in addition, once data are available, uploaded in a cloud that allows to save the recorded sonar data into an online (free) account (https://maps.fishdeeper.com). The recorded data are contained in the bathymetry.csv file, which contains columns for latitude (°), longitude (°), depth (m), temperature (°C), and time (Unix format). Latitude and longitude are recorded with GPS connection at a rate of 1 measurement per second in optimal satellite coverage, while depth and temperature are recorded 15 times /second (Fig. 4). If the data has been recorded in less than 24 h (even if from different location), they are merged into a single file.

If an external GNSS receiver is used, the user must produce a .csv file with the external GNSS antenna points (eventually PPK corrected) with 3 columns including latitudes, longitudes and timestamp (in UNIX format).

## Sonar post-processing pipeline

### Sonar points geolocation reconstruction: “deeper_filler” function

**Fig. 5:**
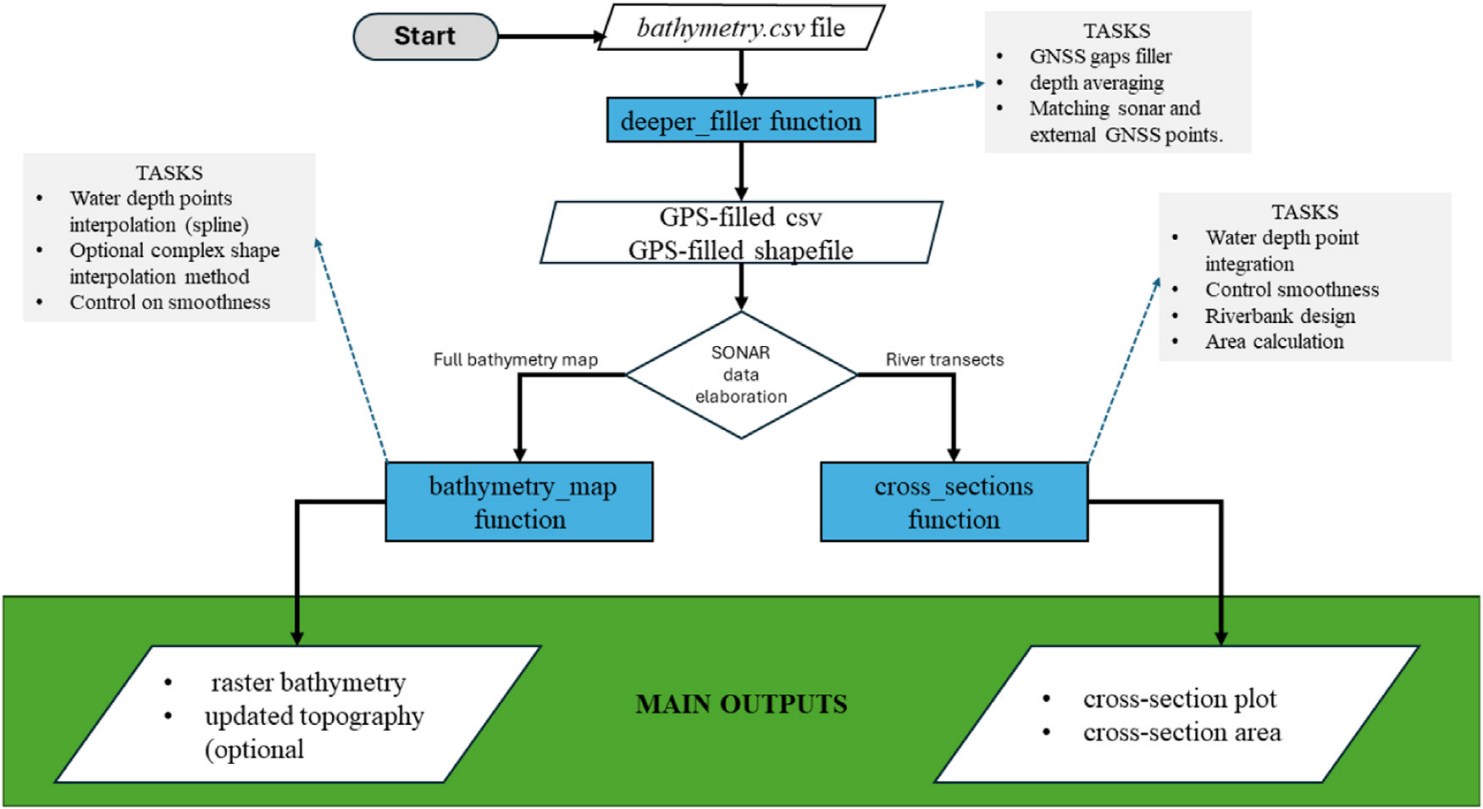
Schematic representation of the “bathymetry.csv” data postprocessing. The available modes are the bathymetry map and cross-section.

The data obtained from the Deeper Chirp+2 sonar (i.e., *bathymetry*.csv file, see section 2) can be ingested in the processing pipeline, that is schematized in Fig. 5.

The *bathymetry*.csv file, once imported in R [7], can be ingested in the base function “deeper_filler”. The “deeper_filler” function main task is to estimate latitudes and longitudes reading missing from the original file due to the different updating timing between sonar and GPS readings (see section 1). The function accepts the following arguments:

1. **bathymetry_path**: path to the original *bathymetry.csv* file.
2. **out_path**: outputs destination path. All outputs are projected in the epgs: 4326 (WGS84) system.
3. **add_proj** (optional); additional projection system for the outputs.
4. **collection_mode**; must be “bathymetry map” (default) or “cross section”.
5. **cross-sectionsID** (optional); only for cross-sections application. Name of the column containing separate ID for each cross-section. If not indicated and collection_mode set to “cross section”, 1 min gap of GNSS measures is interpreted as boundary between consecutive cross sections.
6. **averaging** (empty or integer number); the integer number indicate an area within which a single water depth value is produced by averaging all the contained points.
7. **external_GNSS** (optional); if an external GNSS receiver is coupled with the sonar, indicate the path to the .csv file containing the recorded points coordinates and relative timestamp.

The default *“deeper_filler”* function estimates the missing latitude and longitude coordinates from consecutive existing ones (roughly 1 GNSS position/second, thus every 15 sonar reads, see section 3.1) assuming a linear motion of the sonar in the missing GPS data time (again, ∼1 sec). The missing coordinates are estimated according to the following equations

(2) $$increment=\frac{long,lat_{known_{i}}-long,lat_{known_{i-1}}}{Sonar\;obs}$$

where the $long,lat_{known}$ are the two known points GNSS coordinates that bound unknown position sonar points; Sonarobs is the number of sonar observations between $long,lat_{known}$with unknown GNSS coordinates.

(3) $$long,lat\_sonar_{i}=last\;long,lat_{known}+increment*i$$

Where $long,lat\_sonar_{i}$ are estimated latitude and longitude of the $i^{th}$ sonar observation with unknown GNSS coordinates; $last\;long,lat_{known}$ are the coordinates of the last GNSS measured point.

If mode is set to “cross sections”, (2), (3) are applied separately for each cross-section instance. The averaging option allow user (if not set to False), to specify a number of measurements to be averaged; the averaging is clearly operated on both position (latitude and longitude) and water depth.

In case an external GNSS point file is indicated *deeper_filler* simply match the sonar points with the provided coordinates (eventually PPK corrected) matching the timestamp of the two data and then applying (2), (3) as above.

The outputs obtained from the deeper_filler function are: (I) *filled_bathymetry*.csv file (reference system WGS84) with all the estimated GPS positions; (II) *filled_bathymetry*.shp point file (reference system WGS84); (III) optionally, *filled_bathymetry*.csv and *filled_bathymetry*.shp re-projected in a user-specified reference system.

### Bathymetry map mode: “bathymetry_map” function

**Fig. 6:**
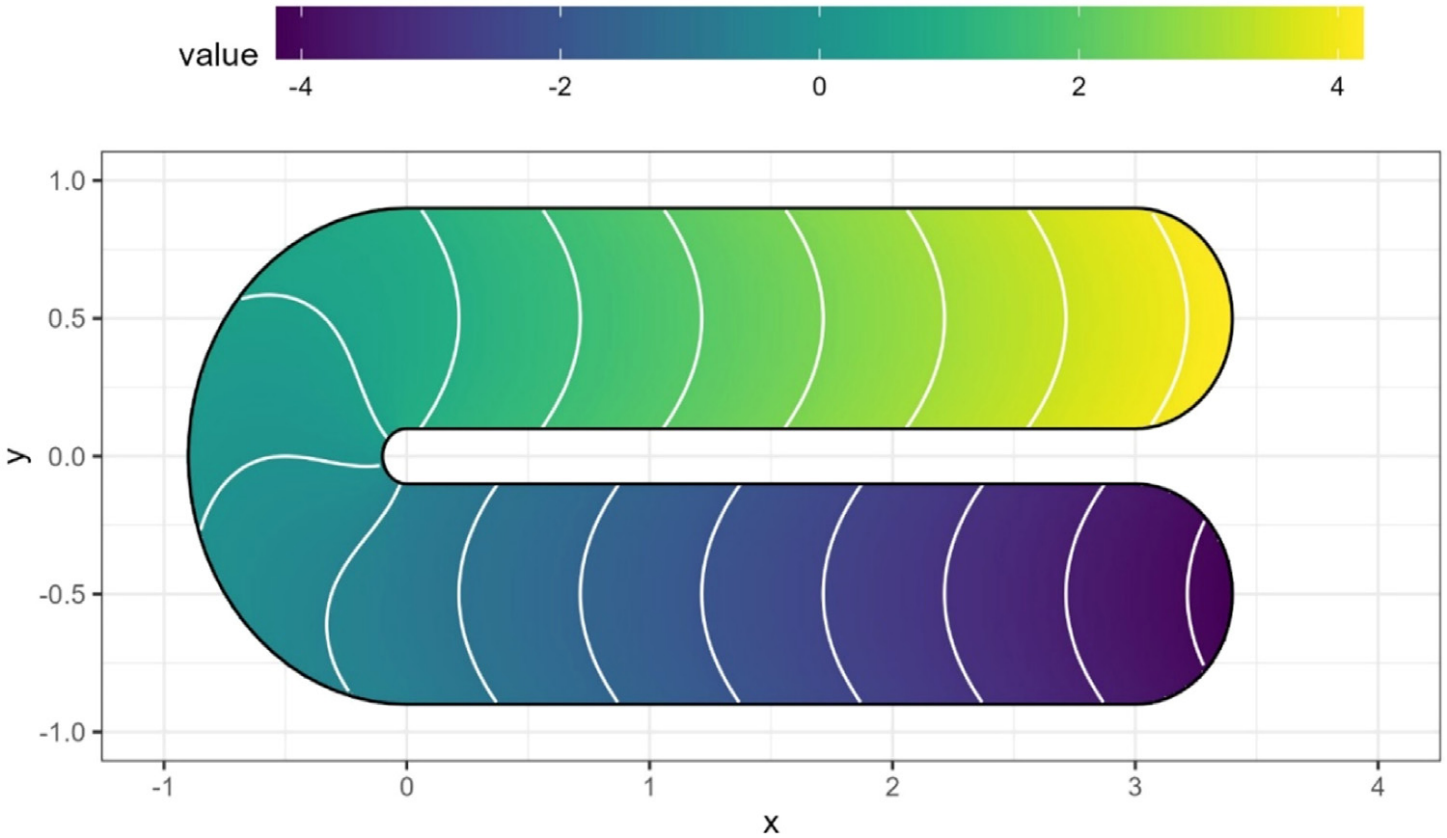
Ramsey's horseshoe function. Closer points (e.g., at the beginning/end of the horseshoe) are more dissimilar than further points.

The bathymetry amp mode is implemented through the *“bathymetry_map”* function. This function is designed specifically to interpolate the bathymetric points producing a bathymetric map. The interpolation is performed through a spline-based method; specifically, a Generalized Additive Model (GAM; [8]) is fitted using a soap film smoothing spline [4]. Bathymetric interpolation, especially for complex shaped areas, is a typical example where the vicinity of the points (i.e., water depth measures) is not a guarantee of points’ similarity; this is known as the smoothing over difficult regions [9] problem. This is well illustrated by the horseshoe Ramsey's function (Fig. 6), where values within the boundary domain vary substantially along the horseshoe path. Thus, the assumption of classical interpolation methods (e.g., Inverse Distance Weighted – IDW) that closer point is more similar than furthest point is incorrect (e.g., in Fig. 6 point close on the “y” axes are substantially dissimilar in their value).

This sort of problem can be found in the bathymetric reconstruction in practical context such as rivers (e.g., meanders area), complex costal zones, areas with presence of “holes” (e.g., islands, emerged areas). The soap film smoothing spline is specifically designed for interpolation within complex region with the possibility to force the interpolation to reach user defined values at the boundary of the polygon (in the case of bathymetric data it can be 0, i.e., no water depth). Thus, it convenient to base this interpolation technique in the *bathymetry_map* function. The *bathymetry_map* function integrate some functionalities form the R packages *mgcv* [8], *terra* [10], *sf* [11] and *stars* [12].

The *bathymetry_map* function accepts the following arguments:

1. **bathymetry_path**; path of the file produced from the deeper_filler function, see section 3.1.
2. **boundary** file; path of the wetted area boundary. The boundary file can be either a *raster*.tiff file or a *polygon.*shp file of the wetted area to be interpolated.
3. **resolution** (optional); the resolution must be indicated only when a *polygon.*shp is provided; it indicates the pixel size (in meter) at which the bathymetry interpolation outputs will be sampled. In case it is provided a *raster*.tiff file, its resolution will be used as interpolation output resolution.
4. **out_path**; otuputs destination path.
5. **overlap_procedure**; True of False (default). See below (Step III)
6. **interpolation_spec** (optional); list of interpolation customization. See below (Step II and step III).
7. **val_method**; it can be an automated process cross-validation method (default) or a set of user-provided validation water depth points. It outputs a set of accuracy metrics to evaluate the interpolation performance (Step V).
8. **add_proj**; by default, the outputs will be produced using the same reference system as the *bathymetry.shp* file; an additional reference file can be specified for re-projected additional output.
9. **DEM** (optional); a *DEM*.tiff file path to be specified if the user need an updated version of the DEM based on the bathymetry produced (Step VI).

Here following is described, step-by step, the procedure implemented in the 2D_bathymerey function to obtain the final bathymetric map. All the steps here listed, are fully automatized and the user just need to provide the inputs specified above (points 1–8). The following figures are based on the bathymetric survey of the channel Sèquia Segona close to campus ETSEAFIV (Escola Tècnica Superior d'Enginyeria Agroalimentària I Forestal I de Veterinària) of the Universitat de Lleida, April 2024. In this case an external GNSS receiver (Xiaomi POCO X3 NFC, Android version 12) has been used to compute the sonar points coordinates after PPK correction using LLEI00ESP base station antenna (EPOS network). The accuracy in the geolocation of sonar points was ∼4 cm.

Step I. *Inputs*

**Fig. 7:**
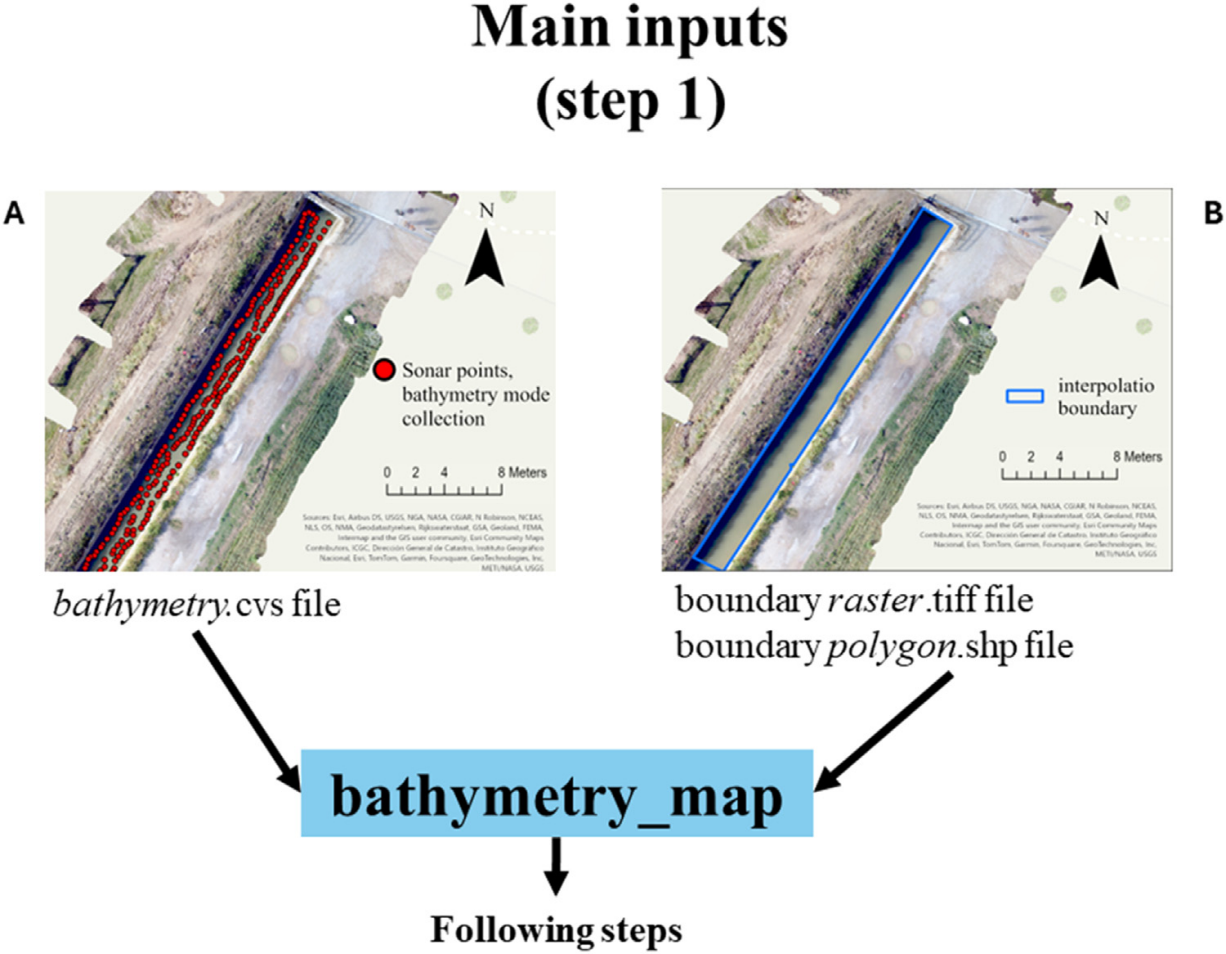
Main inputs file. The bathymetry.shp (A) and the raster.tiff or polygon.shp boundary file (B) are imported in the bathymetry_map function.
Otherwise, if a *polygon.*shp file is provided, the raster version will be produced using the provided pixel size value. The same pixel size will be used to sample the interpolation outputs. Further, must be considered the necessity to produce wetted area file that are temporally closed to the sonar survey, especially for system with substantial variation of the wetted areas (e.g., rivers, tidal area).

The *bathymetry*.shp and the *raster*.tiff or *polygon.*shp boundary files are imported in the bathymetry_map function (Fig. 7). Must be underlined here that the function needs both the vector and the raster form of the boundary; it is able to automatically produce the missing version of the boundary file. If the input is a *raster*.tiff file, it must be a binary raster with the wetted area (i.e., the area within which perform the interpolation) with value of 1 and the non-wetted area as 0. The resolution (i.e., pixel size) of the *raster*.tiff will be used to sample the interpolation outputs. The vector version of the wetted area will be produced through the vectorization of the wetted pixels.

Step II. *Data pre-processing*

**Fig. 8:**
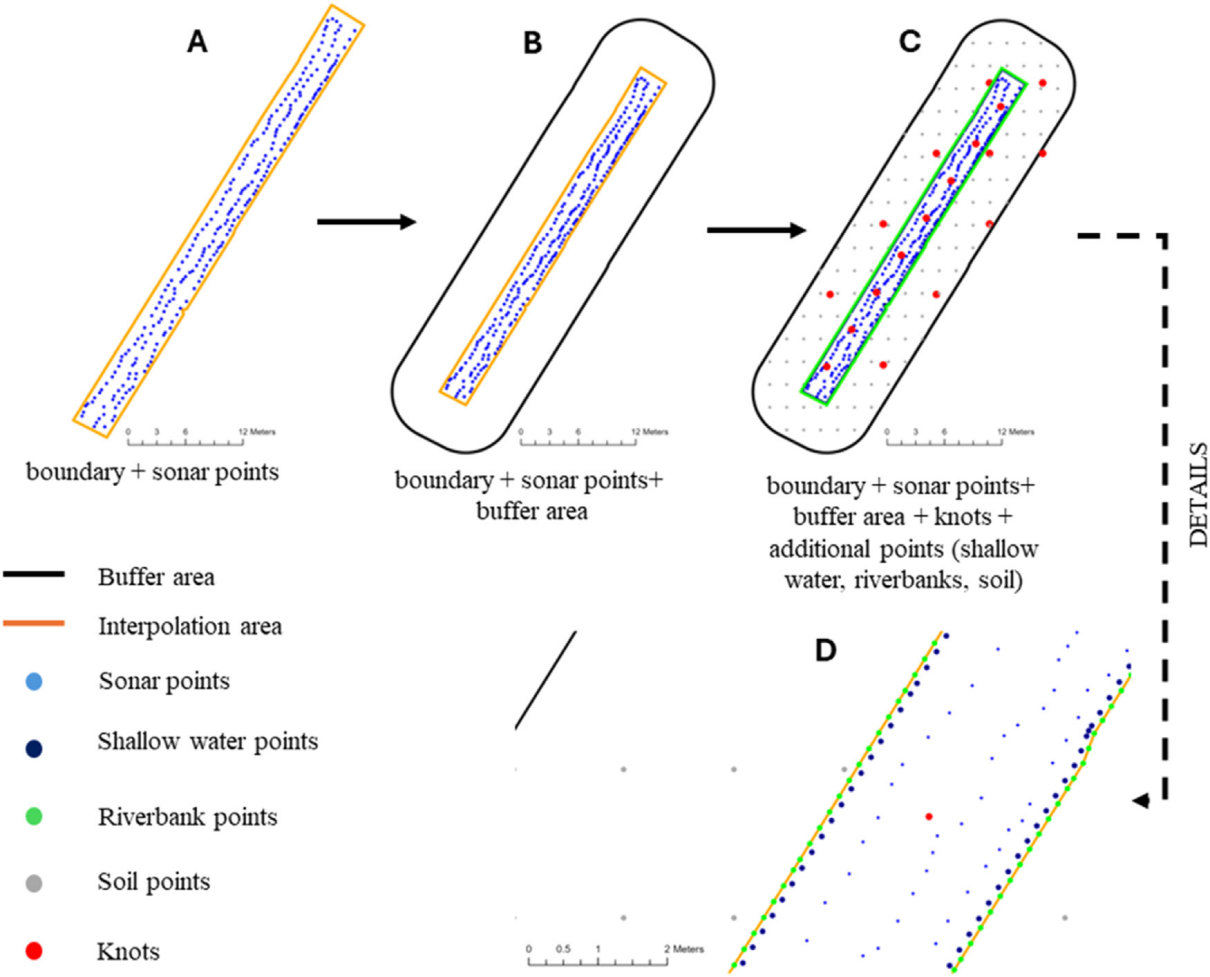
Representation of the data interpolation pre-processing. From the provided sonar points and the interpolation area (A) are computed a buffer zone (black line, B) within which are estimated a number of shallow water points (dark blue, C-D), riverbank points (green, C-D) and soil points (grey, C-D). The last two is attributer a water depth of 0. Further a number of knots (red points, C-D) are located within the interpolation area.
At this stage two customization can be done by the user (function argument 6, see above). Specifically, the user can specify the extension of buffer (in meters) and the water depth of the shallow water points (this can be useful for channels with particular shapes) in form of a single water depth value (i.e., an average value) or in a data frame form with explicit coordinates for each known shallow water depth values.

Once imported the files are checked for projection consistency and that the points provided lay within the wetted area. Following, a buffer is applied around the wetted area and a number of points is produced in the non-wetted areas with a given water depth of 0. A further set of points is produced in the immediate proximity of the boundary itself (30 cm), assuming by default a depth of the water of 0.1 m. We refer to this set of point as shallow water points. The computation of these two sets of point allow to produce the boundary condition for the smoother (Fig. 8). The buffer size is computed by default to be 2 times the average section of the wetted area (computed over 100 random cross-sections). Sonar water depth, shallow water points and non-wetted area points are merged within a unique data frame, where it is indicated if they attain to a wetted (named “W”) or non-wetted (named “T”) domain (information necessary for Step 3).

Step III. *Interpolation model fitting*

The sonar water depth and the boundary depth points produced in Step 2, are fitted with the soap fil smoother thought a Gam. The model takes the form:
  (4) $$Water\;depth=f_{1}\left(x_{i},y_{i}\right)+WT$$
  where $x_{i}$ and $y_{i}$ are the water depth coordinates and $f_{1}$ is a soap-film smoother implemented in a tensor product. WT is a categorical predictor to infirm whether a point attains to the wetted (W) or non-wetted (T) domain.
The number of the knots to fit the smooth function $f_{1}$ is computed separately for wetted and non-wetted areas. Specifically, in the wetted areas the number of knots corresponding to 1/4 of the sonar and shallow water depth points; in the non-wetted area it corresponds to 1/6 of the non-wetted points. In both cases the knots are distribute heavenly through the wetted and non-wetted space; this turns in having a higher knots density in the wetted domain and a lower density in the non-wetted domain (Fig. 8). For high density measurements the user can define the proportion of knots to allow a more efficient computational time. Eq. (2) summary and diagnostic plots (using the *gam.check*() function) are exported in the outputs destination path.

Step IV. *Water depth estimation*

**Fig. 9:**
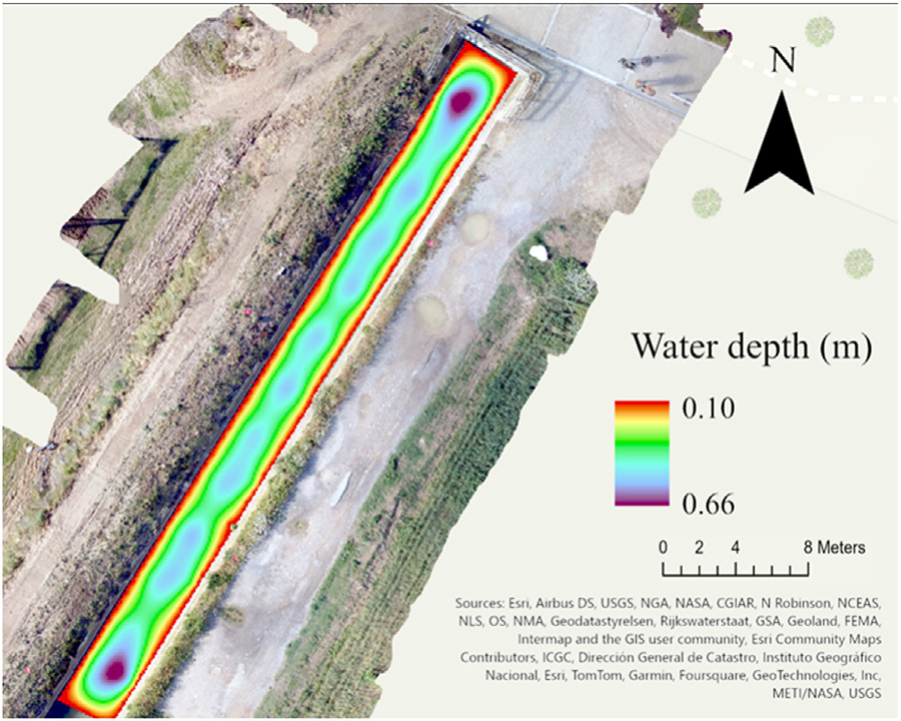
Output bathymetry map raster.
For substantially elongated wetted areas (e.g., extensive river survey with many sonar points) where we expect potentially rapid bathymetric variations (e.g., pools – riffle sequences, confluences morphology, etc.), a sufficient number of knots well distributed thought the area is needed to capture the bathymetric variability. Nonetheless, this will turn into fitting a GAM with potentially a high number of knots, with high computational time. To overcome this issue, bathymetry_map implements the “overlap procedure”. The overlap procedure divides the wetted area to be interpolated in arbitrary sub-areas with length ∼5 times the wetted area cross-section. Each section has an overlap with the adjacent ones. For each section Step II, Step III and Step IV are performed separately and then the predicted raster are mosaicked back to the original full extent.

Once Eq. (4) is fitted the raster version of the wetted and area is produced with a pixel size equal to the one indicated by the user or to the original boundary raster.tiff file (see Step II). Eq. (4) is then used to predict water depth based on the raster pixel coordinates and their classification in to the wetted (W) or non-wetted (T) domain. The estimated bathymetry (Fig. 9) is then exported as a raster in the output destination folder (the default projection is WGS84, but additional projections are added if specified by the user).

Step V. *Accuracy estimation*

The accuracy of the interpolation can be evaluated in two ways. With the default procedure 1/4 of the original sonar water depth points (selected heavenly distributed along the area) are taken out before Step 2 and not involved in any following steps. At this stage they are used to independently validate the interpolation. The second method is by providing an independent set of water depth measures (with known coordinates) on which the accuracy will be estimated. In both cases a series of accuracy metrics are outputted. Specifically, it is reported the absolute Error (AE) for each validation point, the Mean Absolute Error (MAE), the Root Mean Squared Error (RMSE) and linear correlation coefficient (r) between validation and interpolated water depth values. The error terms are computed as follows:
  (5) $$AE=y_{i}-x_{i}$$
  (6) $$MAE=\frac{\sum_{i=1}^{n}AE}{n}$$
  (7) $$RMSE=\sqrt{{\frac{\sum_{i=1}^{n}AE}{n}}^{2}}$$
  (8) $$r=\sqrt{\frac{\left(x_{i}-\bar{x}\right)\left(y_{i}-\bar{y}\right)}{{\left(x_{i}-\bar{x}\right)}^{2}{\left(y_{i}-\bar{y}\right)}^{2}}}$$
  where $y_{i}$ and $x_{i}$ are respectively the validation water depth and the interpolated water depth, $\bar{y}$ and $\bar{x}$ are respectively the mean water depth of the validation and interpolated points and n is the number of validation points.

Step VI. Digital Elevation Model update (optional)

In case the user needs an updated version of an available Digital Elevation Model (DEM) for the area that integrate the river bathymetry (e.g., for hydrological simulation purposes), it is sufficient to indicate the path to the DEM. The DEM will be imported, and the bathymetric values are subtracted from the absolute altitude of the DEM. Must be underlined that the correct alignment between the 2D bathymetry produced by the 2D_batyhmery function and the targeted DEM must be ensured by the user.
To summarize the bathymetry_map function outputs by default a bathymetry.tiff raster file and an accuracy report. Additional outputs are other bathymetry.tiff files with other indicated projections and an updated DEM raster.

### Cross-sections mode: “cross_section” function

The cross-section mode is implemented through the “cross_section” function. It is specifically designed for data collected with the purpose of a 2D representation of the channel and the computation of the section area (e.g., for hydrological purposes). Especially in this case, for accurate estimation of the section area the user must ensure the accuracy of the points position.

The cross_section function accepts the following arguments:

1. **bathymetry_path**; path of the file produced from the deeper_filler function with the “cross_section” mode enabled, see section 3.1.
2. **out_path**; outputs destination path.
3. **riverbank**; path to a .csv file containing 2 columns with the distance between the riverbank and the first sonar data for the right and left riverbank respectively. Right and left are intended to be orographic.
4. **smooth**; True or False (default). If true, the cross section plot and the area are calculated also on a smoothed version of the river bed.

**Fig. 10:**
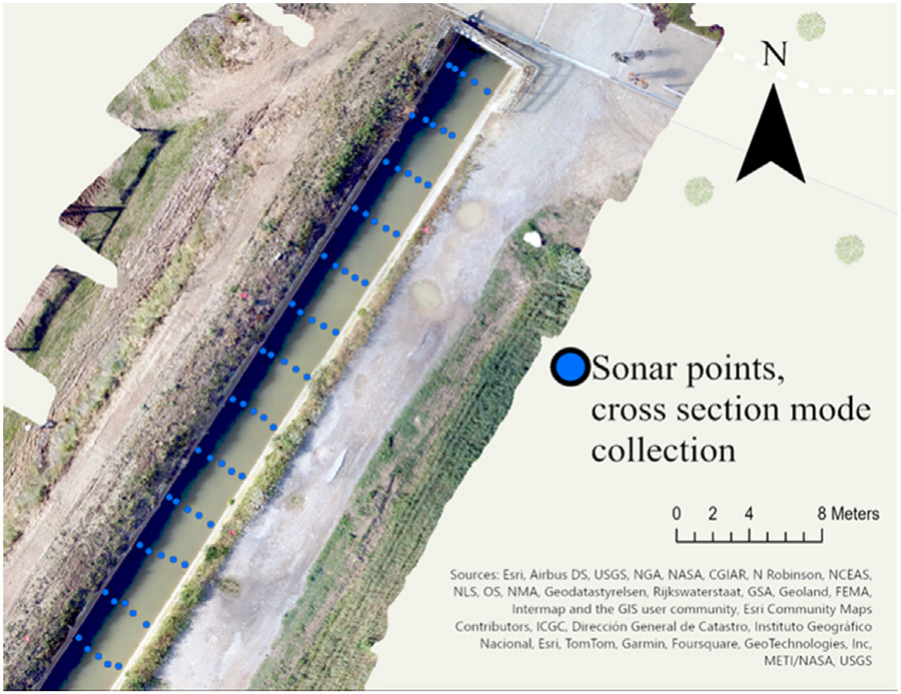
Sonar point location (average point 60 cm).

The cross-section function imports firstly the bathymetry.shp file (Fig. 10) that contains a column to differentiate between different cross sections (see section 3.2). The images and elaboration here reported are produced using an average area of 60 cm (see section 3.2) to match the position of manual measures taken for validation purposes (see *Method validation* section).

**Fig. 11:**
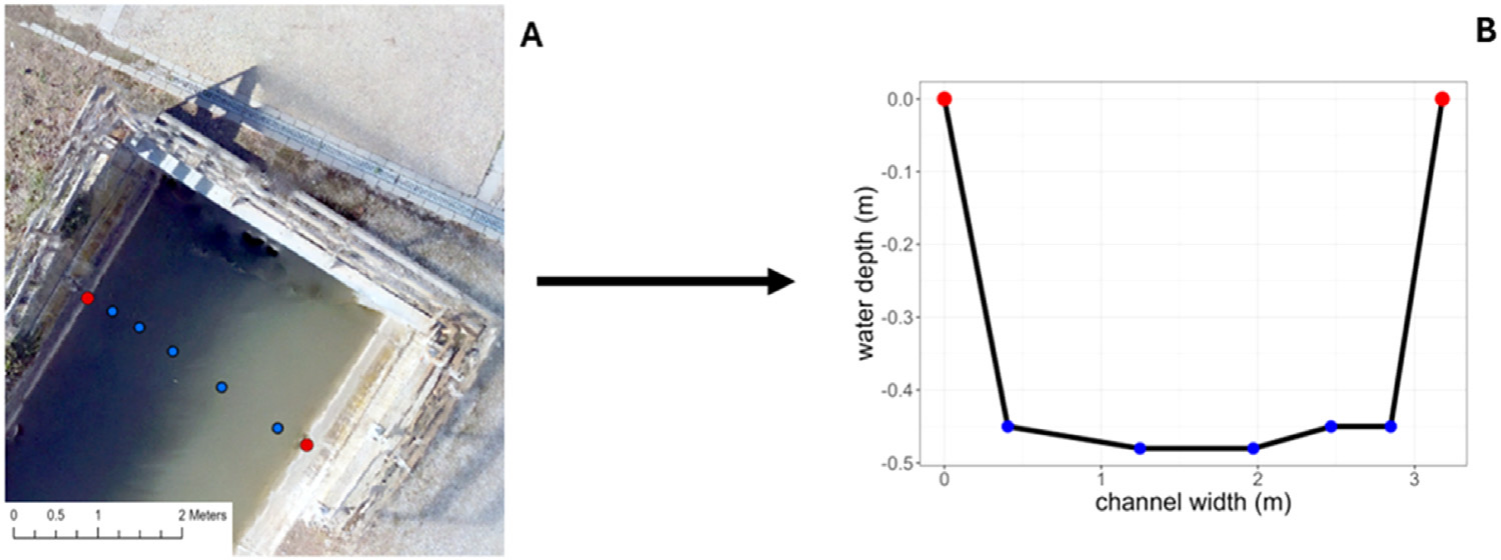
A) Detail of one cross-section with estimated riverbank points based on provided measures (red points), B) Default plot of the cross-section relative to cross-section in A).

Separately for each identified cross section the function locates two riverbank points (one on the right and one on the left side) using the provided distances and assuming their position in line with the last two preceding point in the water. Then it produces a plot and an estimation of the river section area (Fig. 11) based on the sum of the vertical area corresponding to each depth point. In the example of the section reported in Fig. 11, the function calculated an area of 1.31 m^2^ and a relative wetted perimeter of 3.61 m. River section areas and plots are outputted as .csv and .png file respectively.

## Method validation

**Fig. 12:**
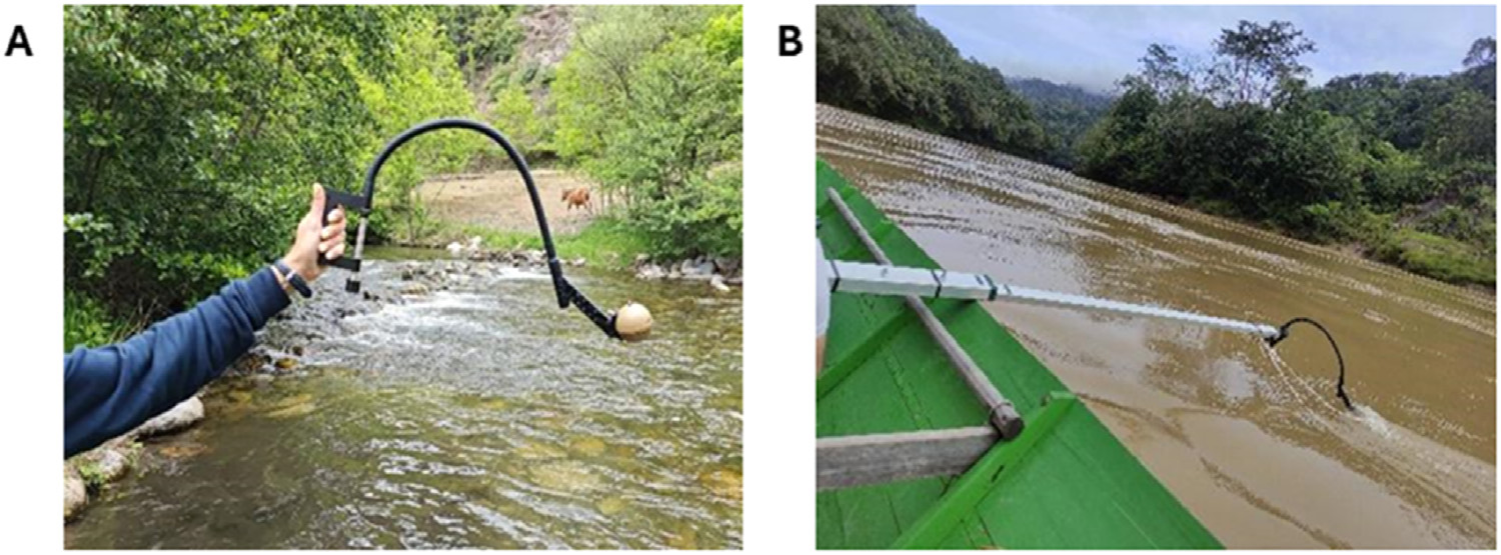
Applications of sonar recording in different data collection setting A) Manually recorded in an alpine river B) attached to an extendable arm on a speedboat during a bathymetric reconstruction in a tropical river.

The methodology here presented allows to produce bathymetry map or cross-sections of river/channel from low-cost sonar data, specifically the Deeper Chirp+2. In addition, it allows to integrate external GNSS receiver to improve sonar point geolocation. All the data elaboration is concentrated in three R-based functions. The methods have been successfully implemented to produce extensive bathymetric maps in tropical context (Redana et al., unpublished) and reconstruct river cross-sections for flow estimation in alpine environment (Carrero-Carralero et al., unpublished). These confirm the robustness of the methodology here proposed, along with the flexibility of the equipment to be deployed in different environmental contexts and experimental setting (Fig. 12).

Nonetheless, the overall accuracy of the bathymetric reconstruction itself largely depends on two aspects: (I) the quality of the original data (i.e., the accuracy of the Deeper Chirp+2 in water depth estimation) and (II) the performance of the interpolation. (I) and (II) concur and interact to define the final accuracy of the bathymetric map estimated with Eqs. (5)–(8) (results provided by the “bathymery_map” function, see 3.2 step V). It can be hard to quantify the specific contribution to the final error of (I) and (II), especially in extensive data collection for large or deep water bodies. The performance of the sonar in the water depth estimation, can vary according to water condition (e.g., turbidity) and experiment setting (e.g., collection speed). Thus we suggest to run some test, prior to the proper data collection, to estimate the accuracy of the Deeper Chirp+2 in environmental and experimental condition as similar as possible to the actual survey. We run this test in the Sèquia Segona channel (see section 3.2), used in this paper as test site for the method on which we based all the figures here reported. For each sonar transect (Fig. 10), water depth was manually measured in 5 points, corresponding to the aggregated sonar point measurement (see section 3.2). We computed the absolute error for each point (AE) and relative error metrics using Eqs. (5)–(8). The resulting Deeper Chirp + 2 MAE = 0.05 m, RMSE = 0.02 m and *r* = 0.91. The outputs of Eqs. (5)–(8) from the “bathymetry_map” functions (thus also considering the errors derived from the interpolation) calculated on the final bathymetric map (Fig. 9) were MAE= 0.03 m, RMSE = 0.01 m and *r* = 0.90.

## Limitations

By combining in three simple R functions a number of new and already existing sub-processes, our method allows an easy and accessible way to produce potentially complex bathymetric maps, automatizing all the process here described. This can represent an alternative, open-source, fully integrated pathway to manage within the same environment all the sonar data workflow. Furthermore, in presence of a large number of data or multiple location, the process can be easily consistently repeated by simply implementing the function within loop not requiring further action for the user. Nonetheless, the automation in R of the procedure comes to a cost of a lower step-by-step and visual control on all the sub-processes internally managed in the functions, that is more typical of classical GIS software - based approaches.

The method itself has been tested and implemented specifically on the Deeper Chirp+2 and its outputs file formats. Even if the outputs file structure is simple, the use of the described methods with other sonar could require some data structure shaping to match the Deeper Chirp+2 format of the bathymetry.csv file (see section 3.1).

We have discussed in the Method validation section the interacting sources of error in the final bathymetric map, deriving from the accuracy of the water depth estimation of the Deeper Chirp+2 and from the interpolation itself. Must be again underlined the necessity to run preliminary tests with the Deeper Chirp+2 on its performance in the water depth estimation in environment and experimental conditions similar to the actual survey. Further we must underly that the Deeper Chirp+2 accuracy in the water depth estimation reported in the Method validation section, has been tested in shallow water conditions. Further test should be carried out to establish the accuracy in deeper water bodies or more complex water quality conditions (e.g., high turbidity, vegetation, etc.). Nonetheless, the accuracy in the water depth estimation do not directly affect the robustness of the data elaboration method here presented.

## Ethics statements

No ethical considerations were required.

## Credit author statement

MR, ECC Conceptualization, Data Curation, Formal Anaysis, Writing - original draft, review and editing, Validation; MR Methodology, Project Administration, Software; ECC Funding acquisition.

## Declaration of competing interest

The authors declare that they have no known competing financial interests or personal relationships that could have appeared to influence the work reported in this paper.

## Data Availability

Data will be made available on request.
